# Growth inhibition of human acute lymphoblastic CCRF-CEM leukemia cells by medicinal plants of the West-Canadian Gwich’in Native Americans

**DOI:** 10.1007/s13659-012-0013-4

**Published:** 2012-02-24

**Authors:** Katharina Deeg, Tolga Eichhorn, Gladys Alexie, Nadine Kretschmer, Kai Andersch, Rudolf Bauer, Thomas Efferth

**Affiliations:** 1 Institute of Pharmacy and Molecular Biotechnology, University of Heidelberg, Heidelberg, Germany; 2 Department of Pharmaceutical Biology, Institute of Pharmacy and Biochemistry, Johannes Gutenberg University, Mainz, Germany; 3 Fort McPerson, Northwest Territories, Canada; 4 Institute of Pharmaceutical Sciences, Department Pharmacognosy, Karl-Franzens University, Graz, Austria; 5 Wilderness International, Dresden, Germany; 6 Stony Plain, Alberta, Canada

**Keywords:** cytotoxicity, herbs, leukemia, medicinal plants, pharmacognosy

## Abstract

The Gwich’in, which belong to the Athapaskan language group of Native Americans live in the borderland between Alaska and Canada. We analyzed 29 medicinal plants of this tribe for their growth inhibitory activity against CCRF-CEM T-cell acute lymphoblastic leukemia (T-ALL) cells. The anti-leukemic activity of these plants has not been investigated as yet. Considering the poor cure rates of some ALL forms, there might be a great potential for medicinal plants as resource for natural products to treat T-ALL. We found that the hexane extracts of three plants revealed considerable growth inhibition on CCRF-CEM cells. The 50% inhibition concentrations (IC_50_) were 6.63 ± 0.03 µg/ml for *Cladina mitis*, 8.65 ± 0.38 µg/ml for *Picea mariana* (needles), and 9.67 ± 1.36 µg/ml for *Artemisia frigida*. Further investigations are required to isolate the active constituents of these plants.

## Introduction

The Athapaskans are a large language family of Native Americans living in Alaska and Canada as well as in the southwest and west coast of USA. The name *Athapaskaw* means "everywhere is pasture and reed" and refers to a region westward of Athabasca Lake (Alberta, Canada). They mainly live from hunting and fishing and in more recent times from fur trade with Europeans. In the borderland between Alaska and Canada, the Kutchin Athapaskans are located which are subdivided in Gwich'in, Loucheux and Takudh. In the present investigation, we analyzed medicinal plants of the Gwich'in. Due to the harsh climate and rough living conditions, medicinal plants played an important role in the everyday life of the Native Americans in these regions.

In the past decades, several papers reported on the ethnobotanical knowledge of these tribes.[1–9] An inventory of medicinal plants of the Carrier, a tribe belonging to the Athapaskan group in North-central British Columbia has been reported.[7] Systematic analyses on the pharmacological activity of medicinal herbs from these tribes are largely missing as of yet. Furthermore, professional timber practices and absorption into modern civilization foster the extinction of medicinal plant species and the loss of indigenous knowledge about medicinal plants.

Interestingly, some characteristics of traditional medicine of these tribes are shared with other traditional medicine systems in other parts of the world, *e.g.* Asia, Africa, or South-America, speaking for an ancient knowledge about medicinal plants tracing back to the early days of manhood as human beings migrated to inhabit our planet. These common features of any traditional medicine include *e.g.* respect for nature and staying in a balance with nature. This is also true for the traditional medicine of the Gwich'in. In their view, diseases result from imbalances between the spiritual and physical world and the task of shamans is to identify such imbalances and treat them by medicinal plants and other means. Plants are collected with a sense of respect and conservation for nature. Single herbs or mixtures are applied as decoctions or infusions for internal use or as ointments, salves and washing solutions for external use.

The Wilderness International Foundation is a non-governmental organization (NGO) helping to preserve wilderness areas and halt widespread environmental damage. To this end, residents and institutions of the endangered areas are involved in the activities of this NGO. The foundation

*To whom correspondence should be addressed. E-mail: efferth@uni-mainz.de maintains a nature preservation project in the Peel River Watershed in North-West Canada, where the Gwich'in and Nacho Nyak Dun "First Nations" are resided. Gaining more knowledge on medicinal plants of the Gwich'in is part of the nature preservation project and was a starting point of the present investigation.

We focused on the cytotoxic activity of medicinal plants of Gwich'in against leukemia cells, because there is a great need for improvement of therapy options worldwide. Acute lymphoblastic leukemia (ALL) is the most common malignancy among children and represents 60% of childhood and 10–20% of adult acute leukemia.[10] The overall ALL incidence rate per year (evaluated from 1975 to 2006) in the USA is 1.32 per 100,000 (http://seer.cancer.gov/csr/1975_2006/results_merged/sect_13_leukemia.pdf).

Untreated ALL is rapidly fatal, with death occurring in weeks to a few months commonly as a consequence of bone marrow failure or space-occupying lesions.[10] In the past decades, the understanding and therapy of childhood ALL has improved a lot, transforming the disease from a rapidly progressive and usually lethal disorder to a disease with an 80% cure rate.[11] Unfortunately, the cure rates of ALL in adults rarely exceed 40%.[11] The poor outcome for adult ALL is probably attributed to drug resistance, poorer tolerance of treatment, and less effective treatment regimens compared with childhood ALL.[11] Furthermore, response to treatment is varying strongly depending on ALL subtype.[12] Although specific treatment approaches may differ for every patient, treatment of ALL generally consists of three phases: remission-induction phase, consolidation phase and continuation therapy to eliminate residual disease.[11,13] Unfortunately, little progress has been made for relapsed and refractory ALL.[14] Furthermore, treatment includes high doses of chemotherapeutics resulting in serious acute and late complications in many patients.[15,16] Thus, there is a significant need for more effective treatments of ALL.

In this context, a promising approach is to utilize the potential of natural products, especially from medicinal plants. Due to the structural diversity of their phytochemical compounds, natural products provide an excellent resource for the identification of novel active agents that may serve as leads and scaffolds for new, more effective drugs in cancer therapy. In the past decades, numerous novel natural compounds with anti-cancer activity have been identified and characterized.[17] Meanwhile, many of them belong to the standard repertoire of cancer chemotherapy.[18] In fact, the relevance of natural products in cancer treatment is emphasized by statistics indicating that natural products and their derivatives account for half of all anti-cancer drugs approved world-wide between the 1940s and 2006.[19] About 75% of plant-derived drugs that are in clinical use attracted attention because of their use in traditional medicines indicating the significance of medicinal plants for the identification of novel anti-cancer drugs.[19] Considering that only 5,000 of 250,000 to 300,000 known plant species have been studied for possible medical application so far, [19] there is still a great treasure waiting to be exploited for drug development.

The objective of the present investigation was to identify medicinal plants that might serve as resources for chemical compounds to treat T-cell ALL. The growth inhibitory activity of medicinal plants was evaluated *in vitro* towards human CCRF-CEM leukemia cells. Our investigation focused on 29 medicinal plants of the Gwich'in, whose anti-cancer activity had not been investigated yet.

## Results and Discussion

**Screening of Extracts from Medicinal Plants.** The results of the screening of plant extracts at a fixed concentration (10 $\mu$g/ml) are summarized in Figure 1. The screening of hexane extracts revealed three hits with an inhibition of leukemia cell growth of more than 60% (Fig. 1A). *Cladina mitis* (CM) had the highest anti-proliferation activity towards CCRF-CEM leukemia cells (nearly 90% inhibition). Needles of *Picea mariana* (PM-N) showed a comparable high cytotoxicity reducing cell viability to approx. 15% of untreated control. Cell viability of CCRF-CEM cells treated with 10 $\mu$g/ml of *Artemisia frigida* (AF) was reduced to 35% of control. Except for *Ledum groenlandicum* (LG), which showed a modest cytotoxic effect (cell viability of less than 60%), all other hexane extracts did not reveal anti-proliferative activity towards CCRF-CEM leukemia cells. Ethanol extracts did not show considerable cytotoxic effects towards CCRF-CEM leukemia cells (Fig. 1B). *Achillea millefolium* (AM), *Aconitum delphiniifolium* (AD) and *Arnica alpina* (AA) rather seemed to induce proliferation of CCRF-CEM leukemia cells. *Solidago spathulata* (SS) showed minimal inhibitory activity. Methanol extracts did also not considerably inhibit leukemia cell growth (Fig. 1C). The screening of ethyl acetate extracts showed comparable results (Fig. 1D). Only *Artemisia frigida* (AF) showed minimal anti-proliferative activity towards CCRF-CEM leukemia cells with a cell viability of about 80% of control.

**Dose-Response Curves of Active Plant Extracts.** To analyze the active extracts in more detail, the dose-response curves of the hexane extracts *Cladina mitis*, *Picea mariana*

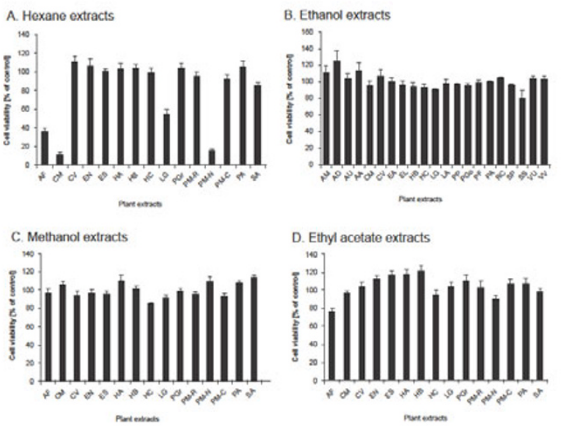

**Figure 1.** Activity of plant extracts towards CCRF-CEM leukemia cells. Cell viability is represented by mean ±SEM of two independent cell viability tests (each with 6 parallel measurements) and is expressed as percentage survival of control (cell growth in medium with solvent). (A) n-hexane extracts; (B) ethanol extracts; (C) methanol extracts; (D) ethyle acetate extracts. The abbreviations for the plants are given in Table 1.

(needles) and *Artemisia frigida* were performed in a dose range from 0.01 to 30 $\mu$g/ml. As shown in Figure 2 and Table 2, the strongest cytotoxic effect was observed in the *Cladina mitis* extract, which had the lowest $IC_{50}$ value (6.63 ± 0.03 $\mu$g/ml). *Picea mariana* (needles) also showed considerable cell growth inhibition ($IC_{50}$ value: 8.65 ± 0.38 $\mu$g/ml). *Artemisia frigida* showed modest activity towards CCRF-CEM leukemia cells ($IC_{50}$ value: 9.67 ± 1.36 $\mu$g/ml).

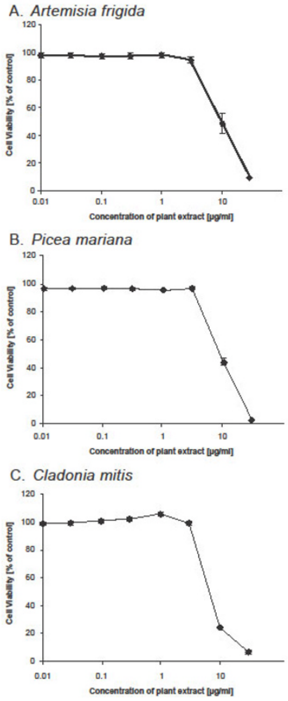

**Figure 2.** Dose response curves of hexane extracts of three medicinal plants of the Gwich'in as measured by the XTT assay. (A) *Artemisia frigida*, (B) *Picea mariana* (needles), and (C) *Cladonia mitis*

The screening of hexane extracts of medicinal plants from the Gwich'in, a Native American tribe from Northwestern Canada, revealed three plants with anti-proliferative effects against CCRF-CEM leukemia cells. *Cladina mitis* (Green Reindeer lichen) has been traditionally applied as tea to treat colds, arthritis, fever and pain in stomach and chest. Furthermore, it has been used as a poultice to relieve pain in arthritic joints and as treatment of tuberculosis. The hexane extract of this plant had the strongest cytotoxic effect in the present investigation. The second active compound was *Picea mariana* (needles), which has traditionally been used as tea against cough and costiveness. The third moderately active plant extract was *Artemisia frigida* (Pasture sage/Prairie sage) that has traditionally been used as tea against severe cold and sore throat and as repellent against mosquitoes, bugs, flea and lice. Extracts of these plants prepared with other solvents (ethanol, methanol, ethylacetate) were inactive. This speaks for specific plant constituents solved in n-hexane that reveal cytotoxicity but not in other solvents.

In general, it can be expected that medicinal plants from traditional medicines contain still unknown natural products with activity towards cancer cells. Examples from the past are the *Vinca* alkaloids from the Madagaskan *Catharanthus roseus* and paclitaxel from the Pacific yew (*Taxus brevifolia*) and related species, *e.g.* *Taxus chinensis*. A plant used by North-American First Nations for the treatment of warts is *Podophyllum peltatum*. Its active constituent, podophylloxotin, served as lead compound for the development of etoposide and teniposide, two clinically well-established anti-cancer drugs. Whether the three plants identified in the present study contain compounds with strong cytotoxicity is still unknown. It is also possible that a combination of different compounds each with weak or intermediate bioactivity act together resulting in a synergistic manner. Previous investigations reported the isolation of secondary metabolites from *Artemisia frigida* and *Picea mariana*.[29–31] Whether these compounds reveal anti-leukemic activity and whether or not synergistic interactions of different constituents are responsible for cytotoxicity needs to be investigated in more detail in the future. Furthermore, whether these plants reveal cytotoxicity to cell lines of solid cancers merits further investigations.

A promising outcome of this study is that it is possible to identify cytotoxic extracts from this panel of plants. This raises the hope that novel phytochemicals may be isolated from medicinal plants of the Gwich'in Native Americans. While medicinal plants have a long tradition among the tribes of Native Americans, a systematic screening and exploitation for therapeutic purposes with modern pharmacological and biological methods has still to be realized.

## Experimental Section

**Cell Line**. The CCRF-CEM cell line is a T lymphoblastic cell line obtained from the peripheral blood of a 4-years old Caucasian female with acute lymphoblastic leukemia[20] and has widely been used as model cell line for T-ALL.[21–23] Human CCRF-CEM leukemia cells were maintained in RPMI 1640 medium (Gibco BRL, Eggenstein) supplemented with 10% fetal bovine serum (Gibco BRL, Trace) and 1% penicillin/streptomycin (Gibco, Invitrogen) in a 5% $CO_2$ atmosphere at 37 °C. Cells were passaged twice a week and medium was changed every two days.

**Plant Extracts**. Plants were collected within a nature conservation expedition of the Wilderness International foundation in the Three Rivers Region (Peel River Watershed) in the border area between the Yukon Territory and the Northwest Territories in the northwest of Canada. A survey of the plants and their traditional applications within the Gwich'in tribes can be seen in Table 1.[24–26] Extraction was performed using ethanol, methanol, n-hexane or ethyl acetate as solvents. Botanical identification was done by two of the authors (K.A. and G.A.) and verified by Prof. Martin Grube (Institute for Plant Science, University of Graz, Austria). Voucher specimens are deposited at the Department for Pharmaceutical Biology, Johannes Gutenberg University, Mainz, Germany.

**Table 1. Survey of medicinal plants from the Gwich'in Native Americans, which were tested for their growth inhibitory activity towards CCRF-CEM leukemia cells. Information about traditional application was obtained from the Gwich'in people during a nature conservation expedition of the Wilderness International foundation and complemented with information from references (Mackinnon et al., 1999; Page, 2004; http://chem.sis.nlm.nih.gov/chemidplus/.).**

| Common Name | Scientific Name | Abbr. | Extract | Traditional Application |
|---|---|---|---|---|
| Alpine Arnica | *Arnica alpina* | AA | EtOH | Preparation of tea; stomach trouble; turgor and effusion |
| Arctic Lupine | *Lupinus arcticus* | LA | EtOH | Seeds toxic, if eaten in large quantities; contains alkaloids |
| Alpine Sweetvetch | *Hedysarum alpinum* | HA | Hex, EtOAc, MeOH | Boiled or roasted roots used as raw vegetables; against diarrhea; as a replacement for licorice |
| Bearberry/ Kinnikinnick | *Arctostaphylos uva-ursi* | AU | EtOH | Berries and petals (in spring) alimental; dried and roasted leaves in tobacco mixtures; leaves used for diuretic tea; halms used for wound healing |
| Black Crowberry | *Empetrum nigrum* | EN | Hex, EtOAc, MeOH | Jam; nutrition; tea made from roots and berries against stomach pain and cold; brewing |
| Black spruce (resin) | *Picea mariana* | PM-R | Hex, EtOAc, MeOH | Chewing gum; wound healing; tea against sore throat; salve greased on the chest against bronchial diseases |
| Black spruce (needles) | *Picea mariana* | PM-N | Hex, EtOAc, MeOH | Tea against cough and costiveness; stomach disease because of tobacco abuse |
| Black spruce (cones) | *Picea mariana* | PM-C | Hex, EtOAc, MeOH | Tea against cold and cough |
| Cloudberry | *Rubus chamaemorus* | RC | EtOH | Berries are vitamin C-containing; nutrition; sap against hives |
| Common Blueberry | *Vaccinium uliginosum* | VU | EtOH | Halms and leaves used in tea against cold; stress repressive |
| Common Snowberry | *Symphoricarpos albus* | SA | Hex, EtOAc, MeOH | Berries are toxic; symptoms include vomiting and diarrhea |
| Cranberry | *Vaccinium vitis-idaea* | VV | EtOH | Nutrition; sap of berries used against trouble with the kidney or digestion and against cold; leaves used in tea against cough; anti-inflammatory |
| Dwarf Scouring Rush | *Equisetum scirpoides* | ES | Hex, EtOAc, MeOH | Raw as nutrition; leaves and halms cooked and used against cold, rheumatism, stomach pain, inflammations and kidney disease |
| Elephant's head | *Pedicularis groenlandica* | PGr | Hex, EtOAc, MeOH | Young blade and roots as raw vegetables or soft boiled; used against fever, contusion and insomnia |
| Fireweed | *Epilobium angustifolium* | EA | EtOH | Sap against skin rash; leaves used as poultice against burning, bee-stings, pain and turgor; nutrition; anti-inflammatory |
| Frog Orchid | *Coeloglossum viride* | CV | EtOH, Hex, EtOAc, MeOH | No known applications |
| Gorman's Beardtongue | *Penstemon gormanii* | PGo | EtOH | Used against toothache |
| Green Reindeer lichen | *Cladina mitis* | CR | EtOH, Hex, EtOAc, MeOH | Used in medicinal tea to treat colds, arthritis, pain in stomach and chest and fever; poultice to relieve pain in arthritic joints; treatment of tuberculosis |

| | | | | |
|---|---|---|---|---|
| Labrador Tea | *Ledum groenlandicum* | LG | EtOH, Hex, EtOAc, MeOH | Tea made of leaves and blossoms against fatigue and stomach ache; evacuent; eases breathing for people with tuberculosis and other respiratory ailments |
| Meadow Alumroot | *Heuchera chlorantha* | HC | EtOH, Hex, EtOAc, MeOH | Poultice made of roots used for wound healing and to stop bleeding |
| Mountain Monkshood | *Aconitum delphiniifolium* | AD | EtOH | Highly toxic; contains alkaloide aconitine; in little doses used against rheumatism and cold |
| Northern Grass of Parnassus | *Parnassia palustris* | PP | EtOH | Sedative; abatement of spasm |
| Northern Sweetvetch | *Hedysarum boreale* | HB | EtOH, Hex, EtOAc, MeOH | Roots are chewed, sweetish taste |
| Shrubby Cinquefoil | *Potentilla fruticosa* | PF | EtOH | No medical application; used for cushion |
| Spikelike Goldenrod | *Solidago spathulata* | SS | EtOH | Contains gum in the sap |
| Pink Wintergreen | *Pyrola asarifolia* | PA | EtOH, Hex, EtOAc, MeOH | Used against rheumatism; wound healing; analgesic; used in tea against cough and renal and liver disorders |
| Prairie Sage/ Pasture Sage | *Artemisia frigida* | AF | Hex, EtOAc, MeOH | Inhalation against cold; Tea against severe cold and sore throat; used against mosquitoes, bugs, flea and lice; anti-inflammatory |
| Willow herb | *Epilobium montanum* | EM | EtOH | Young plants without blossoms for nutrition |
| Yarrow | *Achillea millefolium* | AM | EtOH | Used in tea against cough and nosebleed; alleviates skin irritation and sunburn; against insect bites; stops bleeding of wounds; roots against toothache; anti-inflammatory; anticonvulsant |

**Table 2. 50% Inhibitory concentration ($IC_{50}$) values of CCRF-CEM leukemia cells for *Artemisia frigida*, *Picea mariana* (needles) and *Cladina mitis* extracts. $IC_{50}$ values were calculated from dose-response curves by logarithmic regression. Tests were performed at a dose range from 0.01 to 30 $\mu$g/ml.**

| Scientific name | Common Name | $IC_{50}$ value [$\mu$g/ml] |
|---|---|---|
| ***Artemisia frigida*** | Pasture Sage/Prairie sage | 9.67 ± 1.36 |
| ***Picea mariana*** | Black spruce (needles) | 8.65 ± 0.38 |
| ***Cladina mitis*** | Green reindeer lichen | 6.63 ± 0.03 |

**XTT Proliferation Assay**. The toxicity of compounds was determined by means of the Cell Proliferation Kit II (Roche Diagnostics, Mannheim, Germany). This test is based on the cleavage of the yellow 2,3-bis[2-methoxy-4-nitro-5-sulfo-phenyl]-2H-tetrazolium-5-carboxanilide inner salt (XTT) by ubiquitous dehydrogenases leading to the formation of an orange formazan dye.[27] The amount of dye is commensurate to the number of metabolically active cells. The assay was performed as previously described.[28]

## Acknowledgments

We kindly acknowledge the support of the Gwich'in people (especially the team of Gladys Alexie from Fort McPherson, Northwest Territories, Canada) and of our internship students (Julia Schuler, Jakob Winter, Hannes Holtermann) from Dresden, Germany, who visited our laboratory and helped us to perform the experiments.

**Open Access** This article is distributed under the terms of the Creative Commons Attribution License which permits any use, distribution, and reproduction in any medium, provided the original author(s) and source are credited.
